# Characterization of Diarrheagenic *Escherichia coli* Isolated in Organic Waste Products (Cattle Fecal Matter, Manure and, Slurry) from Cattle’s Markets in Ouagadougou, Burkina Faso

**DOI:** 10.3390/ijerph14101100

**Published:** 2017-09-22

**Authors:** Evariste Bako, Assèta Kagambèga, Kuan Abdoulaye Traore, Touwendsida Serge Bagre, Hadiza Bawa Ibrahim, Soutongnooma Caroline Bouda, Isidore Juste Ouindgueta Bonkoungou, Saidou Kaboré, Cheikna Zongo, Alfred Sababenejo Traore, Nicolas Barro

**Affiliations:** 1 Laboratoire de Biologie Moléculaire d’Epidémiologie et de Surveillance des Bactéries et Virus Transmis par les Aliments (LaBESTA), Centre de Recherche en Sciences Biologiques, Alimentaires et Nutritionnelles (CRSBAN), Université Ouaga I Prof Joseph KI-ZERBO, 03 BP 7021 Ouagadougou 03, Burkina Faso; kagambega.asseta@gmail.com (A.K.); kuanbauer@yahoo.fr (K.A.T.); sergebagre@gmail.com (T.S.B.); hadizabawa@yahoo.fr (H.B.I.); boudacaroline@gmail.com (S.C.B.); ouindgueta@gmail.com (I.J.O.B.); saidoukabore@gmail.com (S.K.); barronicolas@yahoo.fr (N.B.); 2 Institut de Sciences, 01 BP 1757 Ouagadougou 01, Burkina Faso; 3 Direction de la Nutrition, Ministère de la Santé, 03 BP 7068 Ouagadougou 03, Burkina Faso; 4 Laboratoire de Biochimie et d’Immunologie Appliquée (LABIA), Centre de Recherche en Sciences Biologiques Alimentaires et Nutritionnelles (CRSBAN), Université Ouaga I Professeur Joseph KI-ZERBO, 03 BP 7131 Ouagadougou 03, Burkina Faso; zcheik@yahoo.fr; 5 Centre de Recherche en Sciences Biologiques Alimentaires et Nutritionnelles (CRSBAN), Université Ouaga I Professeur Joseph KI-ZERBO, 03 BP 7131 Ouagadougou 03, Burkina Faso; astraore@univ-ouaga.bf

**Keywords:** manure, cattle fecal matter, STEC, ETEC, EAEC, slurries, cattle market, diarrheal diseases, environment sanitation, public health

## Abstract

Cattle farming can promote diarrheal disease transmission through waste, effluents or cattle fecal matter. The study aims to characterize the diarrheagenic *Escherichia coli* (DEC) isolated from cattle feces, manure in the composting process and slurry, collected from four cattle markets in Ouagadougou. A total of 585 samples (340 cattle feces, 200 slurries and 45 manures in the composting process) were collected from the four cattle markets between May 2015 and May 2016. A multiplex Polymerase Chain Reaction (PCR), namely 16-plex PCR, was used to screen simultaneously the virulence genes specific for shiga toxin-producing *E. coli* (STEC), enteropathogenic *E. coli* (EPEC), enterotoxigenic *E. coli* (ETEC), enteroinvasive *E. coli* (EIEC) and enteroaggregative *E. coli* (EAEC). DEC was detected in 10.76% of samples. ETEC was the most prevalent (9.91%). STEC and EAEC have been observed with the same rate (0.51%). ETEC were detected in 12.64% of cattle feces, in 6.66% of manure in the composting process and in 5% of slurry. STEC were detected in 0.58% of cattle feces and in 2.22% of manure in the composting process. EAEC was detected only in 1% of slurry and in 2.22% of manure in the composting process. ETEC strains were identified based on *estIa* gene and/or *estIb* gene and/or *elt* gene amplification. Of the 58 ETEC, 10.34% contained *astA*, 17.24% contained *elt*, 3.44% contained *estIa* and 79.31% contained *estIb*. The two positive EAEC strains contained only the *aggR* gene, and the third was positive only for the *pic* gene. The results show that effluent from cattle markets could contribute to the spreading of DEC in the environment in Burkina Faso.

## 1. Introduction

Fecal contamination is one of the primary contributory factors to the persistence of diarrheagenic pathogens in the environment and contributes to the contamination of food crops and water sources. Intensive cattle farming through cattle fecal matter and effluents contributes to the spreading of these diarrheagenic pathogens in the environment [1]. 

Healthy asymptomatic food animals may carry zoonotic pathogens and represent a principal reservoir of diarrheagenic *Escherichia coli* (DEC), which may enter the food chain via the weak points in the hygiene practices of the slaughter process [2,3].

Traditionally, *E. coli* has been considered a harmless, commensal bacterium. However, several diarrheagenic pathotypes have been recognized based on virulence properties and the mechanisms of pathogenicity [4]. The five main pathotypes of DEC are enteroinvasive *E. coli* (EIEC), enteropathogenic *E. coli* (EPEC), shiga toxin-producing *E. coli* (STEC), enterotoxigenic *E. coli* (ETEC) and enteroaggregative *E. coli* (EAEC) [5,6]. Other authors call these organisms “enteroaggregative, verotoxin-producing *E. coli*.” STEC produces toxin (shiga toxin) encoded by the *stx*_1_ or *stx*_2_ genes or their variants. In addition to shiga toxin (*stx*) gene(s), STEC strains often carry the *eae* gene, encoding the adherence factor intimin. They have also additional virulence genes such as enterohemolysin (EHEC-*hly*) [7]. STEC can cause gastroenteritis, which may be complicated by hemorrhagic colitis or hemolytic-uremic syndrome (HUS), the main cause of acute renal failure in children [8]. EPEC produces characteristic histopathology known as attaching and effacing (A/E) on intestinal cells [9] EPEC is further divided into two subtypes, typical (tEPEC) and atypical (aEPEC), depending on the presence or absence of the EPEC adherence factor (EAF) plasmid and the *bfpB* gene [6,9]. Strains of aEPEC occur most frequently in developed countries, whereas tEPEC is the leading cause of infantile diarrhea in developing countries [10]. ETEC produces heat-labile (LT) and or heat-stable (ST) enterotoxin and is an important cause of diarrhea in infants and travelers, particularly in developing countries [11]. EIEC is associated with invasive, bloody diarrhea resembling that caused by *Shigella* spp. Invasion is mediated by the genes located in virulence plasmid pINV encoding, for example *Ipa* proteins and their transcription regulator *invE* [6,12]. EAEC harbors the mechanism for the aggregative-adherence pattern mediated by aggregative adhesive fimbriae. It is increasingly recognized as a diarrheal pathogen in developing countries [13].

Burkina Faso is a developing country where cattle farming contributes significantly to the economy [14]. Some localized areas serve as cattle markets to help breeders to maximize their profits [15]. Large quantities of waste (fecal matter, manure and manure slurry (liquid waste)) from these markets are directly discharge in the environment without any treatment note far residential areas or applied as fertilizer to land used for silage, grazing or cultivation [16,17,18], what often cause direct contact between humans and these cattle organic waste.

Previous study conducted in Burkina Faso on food animals concluded that cattle are a reservoir for several DEC [19]. Available study showed that DEC are the most predominant pathogens associated with acute diarrhea in children in Burkina Faso [20,21,22].

Despite significant investments in the prevention of diarrheal-related morbidity and mortality, diarrhea remains one of the leading sources of under-five years of age mortality (U5M) worldwide, leading to more than 2100 U5M deaths daily [23]. Among these deaths, more than three-quarters occur mostly in poor and less developed countries of the world [24] with 42% in Sub-Saharan Africa [25].

About 88% of diarrhea-associated deaths are attributable to a lack of safe water, inadequate sanitation and insufficient hygiene [23].

Unless appropriately processed, manure is a potential biohazard capable of transmitting infective agents, including DEC, to both humans and animals [16,17,26,27]. Frequently, DEC is released into the environment through cattle manure [28]. Pathogens may be transported via sediments to vast geographical regions resulting in an increased prevalence in the environment [29,30]. However, the dynamics and routes of the spread of pathogens in farm environments are also poorly understood.

The aim of this study was to characterize diarrheagenic *E. coli* (DEC) strains isolated from cattle feces, manure in the composting process and slurry purchased at four cattle markets located in Ouagadougou, and thus, it provides useful background information to improve manure management practices in urban and peri urban areas for future environmental safety and public health surveillance programs.

## 2. Materials and Methods

### 2.1. Study Design and Sampling Sites 

**Figure 1:**
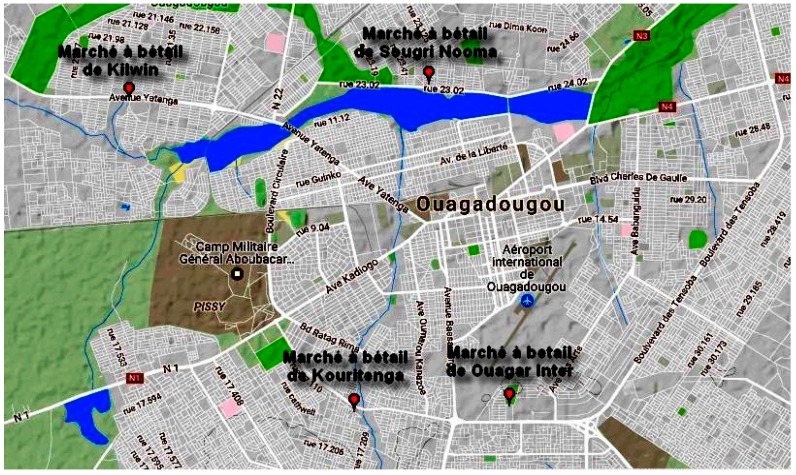
Map of Ouagadougou with the sampling sites. Source: Google Map.

The study was carried out on 4 cattle markets. These markets are respectively the cattle market of Kilwin (12°23′367″ N; 1°35′019″ W), the cattle market of Sougre Nooma (12°23′540″ N; 1°31′836″ W), the cattle market of Ouaga Inter (12°20′156″ N; 1°30′995″ W) and the cattle market of Kouritenga (12°20′078″ N; 1°32′623″ W) (Figure 1). This choice was motivated by the fact that these markets are terminals or consumer markets, where the confluence of a significant number of animals is remarkable. The typology of these markets means that they are most often located in the city near residential areas and certain surface waters such as dams.

A total of 585 samples of cattle feces (*n* = 340), slurry (*n* = 200) and manure in the composting process (*n* = 45) were collected, between May 2015 and May 2016. Slurry was a mixture of feces, urine, water and straw.

Briefly, 200 g of samples (cattle fecal matter, manure in the composting process, slurry) were collected and placed in sterile plastics bags. The samples of cattle feces were collected freshly when the animal defecated, and slurry and manure in the composting process were collected directly from the soil of the sampling site, using a sterile spatula. The samples were transported to the laboratory under appropriate refrigerated conditions and processed within 2 h after collection.

### 2.2. Escherichia coli Isolation

The isolation of *Escherichia coli* strains was carried out according to the guidelines of ISO 4832: 1951 (F). Briefly, 25 g of each sample were added into 225 mL of buffered peptone water (BPW) (Liofilchem, Teramo, Italy) and homogenized using a stomacher (400 Circulator, Seward, London, UK). This step is followed by cascade dilution, which could often go up to 10^−7^ dilution depending on the type of sample. A loopful of these diluents was streaked onto sorbitol MacConkey agar (Liofilchem, Teramo, Italy) and incubated at 44 °C overnight. After incubation, suspected colonies were confirmed by biochemical tests including indole, methyl red, Voges–Proskauer and citrate [31]. The confirmed colonies were conserved in tubes with 1 mL brain heart infusion broth containing 15% (*v*/*v*) glycerol for further analysis at 4 °C. 

### 2.3. Detection of Diarrheagenic Escherichia coli 

DEC were identified by multiplex-PCR [32]. The fragments of 16 virulence genes were amplified with specific primers (Table 1) as previously described [33]. For DNA extraction, a loopful of bacteria growing on sorbitol MacConkey agar was transferred to an Eppendorf tube with 250 µL water. The mixture was boiled for 10 min and centrifuged for 1 min at 13,000 *g*, and the supernatant was analyzed in the PCR reactions. For PCR, 1 µL of the supernatant (DNA) was added to 19 µL reaction mixture containing 4 µL 5× Master Mix (Solis biodyne, Tartu, Estonia), 13 µL water and PCR primers (1 µL Jenni Mix, 1 µL Muller Mix at the concentrations listed in Table 1). The cycling conditions used in the thermal cycler (TC-412) (Bibby Scientific, Lille, France) were 98 °C for 30 s, 35 cycles of 98 °C for 30 s, 62 °C for 60 s and 72 °C for 90 s, with a final extension at 72 °C for 10 min. Amplified DNA fragments were separated by agarose gel electrophoresis (2% weight/volume), stained with ethidium bromide and visualized under UV light. Reference strains EPEC RH 4283 (E 2348/69, [34]), ETEC RH4266 (ATCC 35401), STEC RH 4270 (ATCC 43895), EIEC RH 6647 (145-46-215; Statens Serum Institute, Copenhagen, Denmark), EAEC IH 56822 (patient isolate [35]) and sterile distilled water were included in each PCR run. The criteria for the determination of a DEC were as follows: for EPEC, the presence of *eaeA* and *escV* and possible additional genes *ent* and *bfpB*, and the absence of *bfpB* indicated atypical EPEC; for ETEC, the presence of *elt* and/or *estIa* or *estIb*; for STEC, the presence of *stx*_1_ and or *stx*_2_ and possible additional genes *eae*, *escV*, *ent* and EHEC-hly; for EIEC, the presence of *invE* and *ipaH*; for EAEC, the presence of *pic* and/or *aggR*. The gene *uidA* was used as a general marker for *E. coli*. The gene *astA* was not specific for a certain pathogroup, so it was not used in the final analysis.

## 3. Results

### 3.1. Prevalence of the Escherichia coli Isolate According to the Effluent

A total of 443 strains of *E. coli* was isolated in this study. The study showed that *E. coli* was present in 95% of cattle feces samples, 50% of slurry samples and 44.44% of manure in the composting process samples (Table 2).

### 3.2. Diarrheagenic Virulence Gene Detection and DEC Prevalence

Multiplex PCR assays were used to detect the selected virulence genes of diarrheagenic *Escherichia coli* among 443 *Escherichia coli* strains (Table 3).

DEC was detected in 10.76% of samples (Table 4). A high rate (9.91%) has been observed with ETEC. STEC and EAEC have been observed with the same rate (0.51%). ETEC were detected in 12.64% of cattle feces, in 6.66% of manure in the composting process and in 5% of slurry. STEC were detected in 0.58% of cattle feces and 2.22% of manure in the composting process. EAEC was detected in 1% of slurry and 2.22% of manure in the composting process. ETEC strains contained *estIa* and/or *estIb* and/or *elt* (Table 3). Of the 58 ETEC, 10.34% contained *astA*, 17.24% contained *elt*, 3.44% contained *estIa* and 79.31% contained *estIb*.

The three positive STEC strains were isolated in 2.22% of manure and 0.58% of cattle feces (Table 4). All positive STEC strains contained the gene *stx*_1_. One of them was positive for the *stx*_1_ gene and the *stx*_2_ gene, and the other was positive for the *stx*_2_ gene (Table 3)*.* EAEC was detected in 1% of slurry and 2.22% of manure in the composting process (Table 4). The two positive EAEC strains contained only the *aggR* gene, and the third was positive only for the *pic* gene (Table 3).

**Figure 2:**
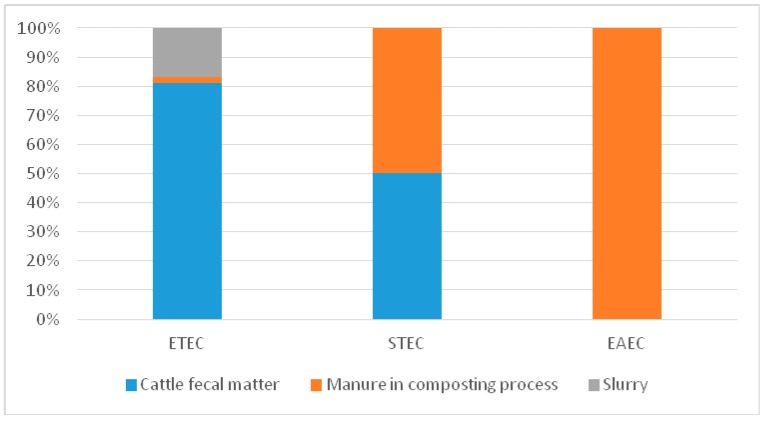
Distribution of diarrheagenic *Escherichia coli* (DEC) in organic waste products from the cattle markets. STEC: shiga toxin-producing *E. coli*; EAEC: enteroaggregative *E. coli*; ETEC: enterotoxigenic *E. coli.*

The distribution of DEC according to the organic waste products of animals (Figure 2) shows that ETEC was present in all organic waste products (cattle fecal matter, manure in the composting process and slurry). STEC was present in manure in the composting process and cattle feces, and EAEC was present in slurry and manure in the composting process.

.

## 4. Discussion

The study investigated the occurrence of five major DEC pathogroups in 585 effluent samples from cattle markets by 16-plex PCR. To our knowledge, this study is the first of its kind in Burkina Faso. In the present study, DEC was detected in 10.76% of samples. In contrast, a lowest prevalence of DEC (7%) was reported in Tanzania by Lupindu et al. (2014) from urban and peri urban livestock cattle feces and soil and water samples [38]. The prevalence of DEC found in this study is worrying because Burkina Faso is facing serious problems in wastewater management due to the demographic explosion and poor urban planning. In Burkina Faso wastewater, manure and slurry are used in crops without being treated beforehand [39]. These practices constitute a high risk for human health. In this study, ETEC 9.91% was the most commonly detected in all effluents purchased at the sites of study. In Brazil, a similar study of the enterovirulence potential of *E. coli* isolates from aquatic environments in Rio de Janeiro has shown that ETEC was present in 5% of agricultural wastewaters [40].

ETEC strains can produce heat-stable (ST) and/or heat-labile (LT) enterotoxins [41]. There are two forms of ST, ST-I (*STa*) and ST-II (*STb*). ST-I has been associated with human infections, and ST-II has been associated with diarrhea in piglets. There are two variants of the human-associated ST-I: STIa (STIp, porcine variant) and STIb (STIh, human variant). The porcine and human variants were originally detected in ETEC strains of porcine and human origin, respectively. However, both ST-I variants can cause disease in humans [41,42]. In our study, we have detected the two variants of heat-stable (ST) enterotoxin. *estIb* (80.70%) was found to be most prevalent, followed by *elt* (17.54%), then *estIa* (3.50%). Furthermore, Kagambèga et al. (2012) had found that *estIb* was most prevalent in food animals’ feces [19]. These findings showed that the spreading of these strains in the environment through waste, effluents or cattle fecal matter constitutes a risk for human health. The persistence and ability of ETEC to survive in effluents like cattle effluent are mostly unknown. However, ETEC could survive for up to three months in freshwater [43] and was able to form biofilms in a wet environment [44].

STEC was detected at a prevalence rate of 0.51% and was present in 0.58% of cattle feces and 2.22% of manure. A similar study has been conducted by Dong et al. (2017) in Gyeonggi Province in Korea, where they obtained a 12.70% prevalence of STEC isolated form cattle farm samples, including feces, ground soil and water [45]. In contrast, Kabiru et al. (2015) have reported 1.37% of STEC in effluent from the slaughterhouse spills in Nigeria [46]. These differences could be explained by the difference in sampling and/or the method used for the isolation of STEC. Indeed, studies have shown that young animals, such as calves and lambs, were the largest excretors of STEC [47], while in general, in Africa, cattle encountered at cattle markets for sale are mostly adults.

Shiga toxin (stx) is the main virulence trait of STEC. There are two types of shiga toxin, *stx*_1_ and *stx*_2_, encoded by the genes *stx*_1_ and *stx*_2_. The mechanism of Stx delivery and trafficking through endothelial cells, such as renal cells, has been thoroughly described by [48].

In this study, the gene *stx_1_* was present in all STEC isolates (100%), and one of them (33.33%) carried both forms of the stx virulence genes (*stx*_1_ and *stx*_2_). In the Eastern Cape Province of South Africa, Iweriebor et al. (2015) obtained stx virulence genes in STEC strains isolated from dairy cattle farms, distributed as follows: *stx*_1_ (38.94%), *stx*_2_ (40%) and two forms of stx virulence genes *stx*_1,_ *stx*_2_ (9.47%) [49]. In Australia, *stx*_1_ alone or in combination with *stx*_2_ was found to be more prevalent than *stx*_2_ alone in STEC from humans, ruminant feces and meat [50]. The variability of stx gene in STEC from the same and or different countries could be explained by the fact that *E. coli* strains have the ability to lose or gain genes easily from their environment. This implies that the clinical signs of a diarrheal disease at STEC in Burkina Faso could arise under various manifestations.

EAEC was detected at a prevalence rate of 0.51% and has been detected in slurry (1%) and manure in the composting process (2.22%). A similar study of the slaughterhouses’ effluent has been reported in the Zaria region, Nigeria [46]. EAEC strains were positive for *aggR* (66.66%) and the gene *pic* (33.33%). Similar results were found in EAEC strains isolated in food animals and humans in Burkina Faso [51,52]. This result confirmed that food animals are the principal source of human DEC, which can be transmitted to humans in different ways.

According to the literature, EAEC are highly adapted to humans, suggesting that the human population is their reservoir [53,54]. Additionally, the isolation of EAEC from animals including cattle and abattoir effluent has been unsuccessfully attempted in different studies [55,56]. 

The distribution of DEC according to the organic waste product of animal origin shows that cattle feces were contaminated by ETEC, STEC and EAEC. A previous study has shown that cattle feces may contain several DEC [19]. Manure in the composting process was contaminated by STEC and ETEC. STEC and ETEC have also been isolated from manure in the United States of America [56]. In general, the ability of these pathogens to adapt and persist in environments is inherent to *E. coli* [57]. Despite the low prevalence noted for these pathotypes, their presence in organic wastes reveals the risk for the population’s health.

## 5. Conclusions

This study demonstrates that organic waste products from cattle markets in Ouagadougou are commonly contaminated by DEC, like ETEC, STEC and EAEC. The presence of these DEC in these effluents (cattle feces, manure in the composting process and slurry) shows that these effluents can constitute a means of human exposition and play an important role in the maintenance of the epidemiological cycle of diarrheal pathotypes of *E. coli* in Burkina Faso. This study shows the need for adequate management of the hygiene of these effluents before any form of recovery or any release of these effluents into the environment, to limit the spread of diarrheal strains of *E. coli* from animals to humans through the environment.

## Figures and Tables

**Table 1:** 16-Plex PCR primers and the virulence genes detected.

Pathotypes	Target Genes	Primer Sequence (5′–3′)	Product Size (bp)	Concentration (µM)	References
STEC, EPEC	*eaeA*	eae-F: TCAATGCAGTTCCGTTATCAGTT eae-R: GTAAAGTCCGTTACCCCAACCTG	482	0.1	[36]
	*escV*	MP3-escV-F: ATTCTGGCTCTCTTCTTCTTTATGGCTG MP3-escV-R: CGTCCCCTTTTACAAACTTCATCGC	544	0.4	[32]
	*ent*	ent-F: TGGGCTAAAAGAAGACACACTG ent-R: CAAGCATCCTGATTATCTCACC	629	0.4	[32]
Typical EPEC	*bfpB*	MP3-bfpB-F: GACACCTCATTGCTGAAGTCG MP3-bfpB-R: CCAGAACACCTCCGTTATGC	910	0.1	[32]
STEC	*EHEC-hly*	hlyEHEC-F: TTCTGGGAAACAGTGACGCACATA hlyEHEC-R: TCACCGATCTTCTCATCCCAATG	688	0.1	[33]
	*stx*_1_	MP4-stx1A F: CGATGTTACGGTTTGTTACTGTGACAGC MP4-stx1A-R: AATGCCACGCTTCCCAGAATTG	244	0.2	[32]
	*stx*_2_	MP3-stx2A-F: GTTTTGACCATCTTCGTCTGATTATTGAG MP3-stx2A-R: AGCGTAAGGCTTCTGCTGTGAC	324	0.4	[32]
EIEC	*ipaH*	ipaH-F: GAAAACCCTCCTGGTCCATCAGG ipaH-R: GCCGGTCAGCCACCCTCTGAGAGTAC	437	0.1	[33]
	*invE*	MP2-invE-F: CGATAGATGGCGAGAAATTATATCCCG MP2-invE-R: CGATCAAGAATCCCTAACAGAAGAATCAC	766	0.2	[37]
EAEC	*aggR*	MP2-aggR-F: ACGCAGAGTTGCCTGATAAAG MP2-aggR-R: AATACAGAATCGTCAGCATCAGC	400	0.2	[32]
	*pic*	MP2-pic-F: AGCCGTTTCCGCAGAAGCC MP2-pic-R: AAATGTCAGTGAACCGACGATTGG	111	0.2	[32]
	*astA*	MP2-astA-F: TGCCATCAACACAGTATATCCG MP2-astA-R: ACGGCTTTGTAGTCCTTCCAT	102	0.4	[32]
ETEC	*elt*	MP2-LT-F: GAACAGGAGGTTTCTGCGTTAGGTG MP2-LT-R: CTTTCAATGGCTTTTTTTTGGGAGTC	655	0.1	[32]
	*estIa*	MP4-STIa-F: CCTCTTTTAGYCAGACARCTGAATCASTTG MP4-STIa-R: CAGGCAGGATTACAACAAAGTTCACAG	157	0.4	[32]
	*estIb*	MP2-STI-F: TGTCTTTTTCACCTTTCGCTC MP2-STI-R: CGGTACAAGCAGGATTACAACAC	171	0.2	[32]
*E. coli*	*uidA*	MP2-uidA-F: ATGCCAGTCCAGCGTTTTTGC MP2-uidA-R: AAAGTGTGGGTCAATAATCAGGAAGTG	1487	0.2	[32]

STEC, shiga toxin-producing *Escherichia coli*; EPEC, enteropathogenic *E. coli*; EIEC, enteroinvasive *E. coli*; EAEC, enteroaggregative *E. coli*; ETEC, enterotoxigenic *E. coli*.

**Table 2:** Prevalence of *Escherichia coli* in organic waste products from cattle markets.

Type of Effluent	*Escherichia coli* Isolates	
	Number	%
Manure in the composting process (*n* = 45)	20	44.44
Cattle feces (*n* = 340)	323	95
Slurry (*n* = 200)	100	50
Total (*n* = 585)	443	75.72

% = Prevalence of isolation.

**Table 3:** Virulence genes detected by 16-plex PCR in *Escherichia coli* isolates and in the six control strains.

*E. coli* Pathogroups	Virulence Gene															
	*eae*	*esCv*	*ent*	*bfp*	*EHEC-hly*	*stx_1_*	*stx_2_*	*ipaH*	*invE*	*aggR*	*Pic*	*astA*	*elt*	*estIa*	*estIb*	*uidA*
STEC	+	+	+	-	+	+	+	-	-	-	-	-	-	-	-	+
EPEC	+	+	+	-	-	-	-	-	-	-	-	-	-	-	-	-
ETEC	-	-	-	-	-	-	-	-	-	-	-	+	+	-	+	+
EAEC	-	-	-	-	-	-	-	-	-	+	+	+	-	-	-	+
EIEC	-	-	-	-	-	-	-	-	-	+	+	-	-	-	-	+
Number of virulence genes detected among *Escherichia coli* strains																
STEC	-	-	-	-	-	3	1	-	-	-	-	-	-	-	-	3
ETEC	-	-	-	-	-	-	-	-	-	-	-	6	10	2	46	60
EAEC	-	-	-	-	-	-	-	-	-	2	1	-	-	-	-	6

+ = positive; - = none; EPEC: enteropathogenic *E. coli*; STEC: shiga toxin-producing *E. coli*; EHEC: enterohemorrhagic *E. coli*; EIEC: enteroinvasive *E. coli*; EAEC: enteroaggregative *E. coli*; ETEC: enterotoxigenic *E. coli*.

**Table 4:** Prevalence of diarrheagenic *Escherichia coli* (DEC) in organic waste products from cattle markets.

*E. coli* Pathogroups	Samples			Total (*n* = 585)
	Cattle Feces (*n* = 340)	Slurry (*n* = 200)	Manure in the Composting Process (*n* = 45)	
Any DEC	45 (13.23%)	13 (6.5%)	5 (11.11%)	63 (10.76%)
STEC only	2 (0.58%)	0	1 (2.22%)	3 (0.51%)
ETEC only	43 (12.64%)	11 (5%)	3 (6.66%)	58 (9.91%)
EAEC only	0	2 (1%)	1 (2.22%)	3 (0.51%)

0: none; EPEC: enteropathogenic *E. coli*; STEC: shiga toxin-producing *E. coli*; EHEC: enterohemorrhagic *E. coli*; EIEC: enteroinvasive *E. coli*; EAEC: enteroaggregative *E. coli*; ETEC: enterotoxigenic *E. coli*; DEC = diarrheagenic *Escherichia coli*.
